# Mouse *Idh3a* mutations cause retinal degeneration and reduced mitochondrial function

**DOI:** 10.1242/dmm.036426

**Published:** 2018-12-18

**Authors:** Amy S. Findlay, Roderick N. Carter, Becky Starbuck, Lisa McKie, Klára Nováková, Peter S. Budd, Margaret A. Keighren, Joseph A. Marsh, Sally H. Cross, Michelle M. Simon, Paul K. Potter, Nicholas M. Morton, Ian J. Jackson

**Affiliations:** 1 MRC Human Genetics Unit, Institute of Genetics and Molecular Medicine, University of Edinburgh, Crewe Road, Edinburgh EH4 2XU, UK; 2 Molecular Metabolism Group, Centre for Cardiovascular Sciences, Queens Medical Research Institute, University of Edinburgh, Edinburgh EH16 4TJ, UK; 3 MRC Mammalian Genetics Unit, MRC Harwell Institute, Harwell Campus, Oxfordshire OX11 0RD, UK; 4 Roslin Institute, University of Edinburgh, Edinburgh EH25 9RG, UK

**Keywords:** Krebs cycle, Mouse model, Retinitis pigmentosa

## Abstract

Isocitrate dehydrogenase (IDH) is an enzyme required for the production of α-ketoglutarate from isocitrate. IDH3 generates the NADH used in the mitochondria for ATP production, and is a tetramer made up of two α, one β and one γ subunit. Loss-of-function and missense mutations in both *IDH3A* and *IDH3B* have previously been implicated in families exhibiting retinal degeneration. Using mouse models, we investigated the role of IDH3 in retinal disease and mitochondrial function. We identified mice with late-onset retinal degeneration in a screen of ageing mice carrying an ENU-induced mutation, E229K, in *Idh3a*. Mice homozygous for this mutation exhibit signs of retinal stress, indicated by GFAP staining, as early as 3 months, but no other tissues appear to be affected. We produced a knockout of *Idh3a* and found that homozygous mice do not survive past early embryogenesis. *Idh3a^−/E229K^* compound heterozygous mutants exhibit a more severe retinal degeneration compared with *Idh3a^E229K/E229K^* homozygous mutants. Analysis of mitochondrial function in mutant cell lines highlighted a reduction in mitochondrial maximal respiration and reserve capacity levels in both *Idh3a^E229K/E229K^* and *Idh3a^−/E229K^* cells. Loss-of-function *Idh3b* mutants do not exhibit the same retinal degeneration phenotype, with no signs of retinal stress or reduction in mitochondrial respiration. It has previously been reported that the retina operates with a limited mitochondrial reserve capacity and we suggest that this, in combination with the reduced reserve capacity in mutants, explains the degenerative phenotype observed in *Idh3a* mutant mice.

This article has an associated First Person interview with the first author of the paper.

## Abstract (precis)

**Summary:** Here, we show that partial loss-of-function mutations in the *Idh3a* gene lead to retinal degeneration due to compromised mitochondrial function. Complete loss of *Idh3a*, however, is embryonically lethal.

## INTRODUCTION

Mitochondrial diseases are highly diverse: over 220 different genes have been identified that can cause diseases of the mitochondria, and these diseases have a wide range of pathologies (Koopman et al., 2012). Common to many of the diseases, however, are neurological or neuromuscular manifestations, including retinal disease. We have identified a missense mutation in the gene encoding a subunit of the tricarboxylic acid (TCA) cycle enzyme isocitrate dehydrogenase 3 (IDH3) as causing a retinal degeneration phenotype in mice. There are three isocitrate dehydrogenase isozymes in mammals. IDH1 and IDH2 are both homodimers that catalyse the decarboxylation of isocitrate to α-ketoglutarate with the concomitant reduction of NADP to NADPH. IDH1 is cytoplasmic, whereas IDH2 is localised to the mitochondria. No inherited disease has been associated with *IDH1* mutations, but mutations in *IDH2* cause a metabolic disease, D-2-hydroxyglutaric aciduria, with a range of features including epilepsy, hypotonia and other neurological manifestations (Kranendijk et al., 2010). *IDH1* or *IDH2* has been found to be mutated in a high proportion of glioblastomas and other tumours of the nervous system. By contrast, IDH3 is a heterotetramer, composed of two α, one β and one γ subunit, encoded by three genes. IDH3 is located in the mitochondria, where the conversion of isocitrate to α-ketoglutarate is necessary for the TCA cycle to progress and generate NADH, which feeds into oxidative phosphorylation to generate ATP. No recurrent mutations in any of the IDH3 genes have been described in tumours.

A number of patients have been described with a variety of mutations in *IDH3A* (Fattal-Valevski et al., 2017; Pierrache et al., 2017). A patient with the most severe of these, a homozygous missense mutation (p.Pro304His), exhibited neurological defects from birth, possibly due to a failure during development, as well as retinal degeneration. However, the patient showed no symptoms of muscle weakness, typically associated with mitochondrial deficiencies. The remaining patients, with bi-allelic variants, all exhibited childhood onset of retinal degeneration and some had pseudocoloboma of the macula. Pseudocoloboma is not a developmental defect but is caused by degeneration of the retina. Seven different alleles were found in four different families all predicted to be pathogenic, including two that could potentially cause nonsense-mediated decay. The other five, all missense mutations, are anticipated to be damaging. Two families with homozygous mutations in *IDH3B*, and which exhibited non-syndromic retinal degeneration, have also been described (Hartong et al., 2008). None of these patients showed symptoms of mitochondrial dysfunction other than retinitis pigmentosa (RP).

We have identified a missense mutation, E229K, in mouse *Idh3a*, which is associated with late-onset retinal degeneration. In addition, we generated a complete loss-of-function mutation in this gene and show that it is early embryonic lethal. This mutation, when combined with the missense mutation *Idh3a^E229K^*, results in a more rapid retinal degeneration than that in the missense homozygotes. Photoreceptors of the retina have a particularly high energy requirement, and the mitochondria of these cells function at 70-80% of their maximal rate, leaving limited reserve capacity (Kooragayala et al., 2015). This most likely accounts for the sensitivity of the retina to mitochondrial mutations. On the other hand, a loss-of-function mutation in *Idh3b* is viable and has no detectable abnormal phenotype. The retina of these mice are normal up to 6 months of age. We have analysed the mitochondrial function in cells with *Idh3* mutations, and find the maximum and reserve capacity of *Idh3a* mutant cells to be reduced. However, cells lacking IDH3B show no detectable mitochondrial defect.

## RESULTS

**Fig. 1.**
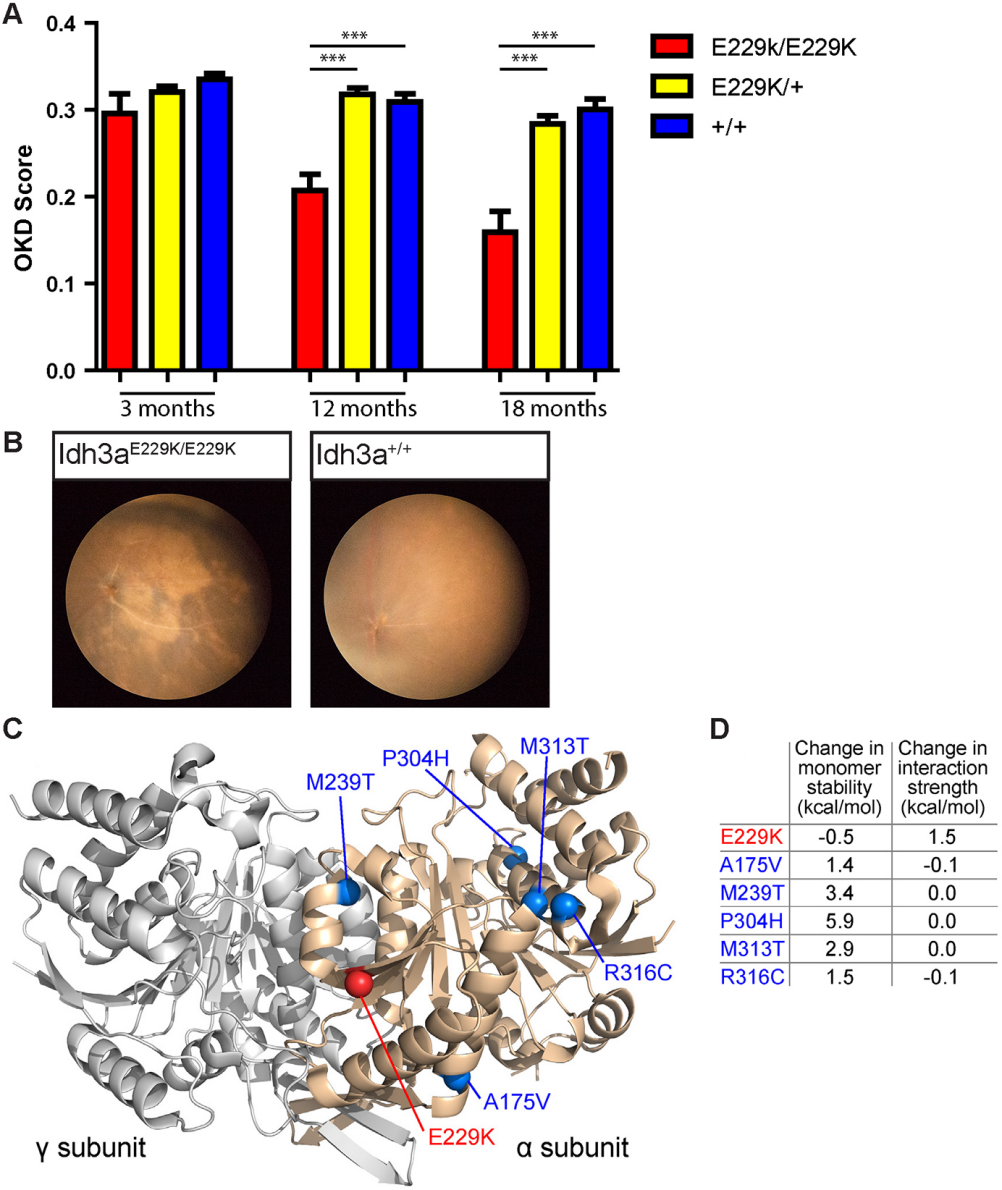
***Idh3a^E229K/E229K^* mice exhibit signs of retinal degeneration.** (A) *Idh3a^E229K/E229K^* mice (red) showed a decline in visual acuity, as determined by optokinetic drum (OKD) score, by 12 months {0.2071±0.01872 c/d [mean±s.e.m., *n*=9, female (F) 4, male (M) 5]} compared with wild-type (blue) [0.3090±0.009441 c/d (mean±s.e.m., *n*=23, F14, M9)] (*P*<0.0001) and heterozygous (yellow) [0.3173±0.007472 c/d (mean±s.e.m., *n*=35, F24, M11)] (*P*<0.0001) mice. This significant decline continued and, by 18 months, homozygous mice (red) had degenerated further [0.1592±0.02396 c/d (mean±s.e.m., *n*=9 F4, M5)] compared with wild-type (blue) [0.3004±0.01223 c/d (mean±s.e.m., *n*=23, F14, M9)] (*P*<0.0001) and heterozygous (yellow) [0.2836±0.009699 c/d (mean±s.e.m., *n*=35, F24, M11)] mice (*P*<0.0001). Unpaired *t*-test, ***P<0.001. (B) Fundus imaging comparing *Idh3a^E229K/E229K^* retina with that of a wild-type littermate; the mutant retina exhibits signs of retinal degeneration, as evidenced by a dark patchy appearance. (C) Structure of the IDH3A:IDH3G heterodimer (PDB ID: 5GRI) with the positions of the E229K mutation identified in this study (red) and previously identified missense mutations (blue) highlighted. (D) Predicted effects of mutations on the stability of the IDH3A monomer and on the strength of the interaction with IDH3G. Higher values indicate greater disruption of protein stability or interaction strength.

Following a screen for N-ethyl-N-nitrosourea (ENU)-induced recessive mutations with an age-dependent phenotype, we identified a mouse line that exhibited loss of vision and retinal degeneration associated with increasing age (Potter et al., 2016). When subjected to a moving visual stimulus within an optokinetic drum (OKD) at 12 months of age, a subset of the littermates in this cross responded with the stereotypical head movement only at ∼0.2 cycles per degree (c/d), compared with >0.3 c/d for phenotypically wild-type littermates. These mice maintained a diminished response at 18 months (Fig. 1A). The same mice had abnormal retina, identified by indirect ophthalmoscopy and documented by fundal imaging at 7 months (Fig. 1B). The dark patches on the retinal images are indicative of retinal degeneration. No other overt phenotypes were detected in the mice up to 18 months of age (Table S1).

Genotyping of the affected littermates in this cross revealed a single common region of homozygosity on chromosome 9, between 50.1 Mb and 74.8 Mb. Analysis of whole-genome sequence of the founder male for this line found two candidate protein coding mutations in the interval, heterozygous in the founder: sorting nexin 33 (*Snx33*) and the α subunit of isocitrate dehydrogenase 3 (*Idh3a*). The *Snx33* mutation was not present in the mutant line, presumably because it is on the unaffected chromosome. We considered the missense mutation, E229K, in *Idh3a* to be the likely causative mutation, as human patients with mutations in either *IDH3A* or *IDH3B* exhibit retinal degeneration (Fattal-Valevski et al., 2017; Hartong et al., 2008; Pierrache et al., 2017). To confirm *Idh3a* as the causative gene, we used CRISPR/Cas9 to introduce a loss-of-function mutation. We generated an insertion of 5 bp into exon 7 of the gene, c.702_703InsGTACT, termed *Idh3a^em1Jkn^*, abbreviated herein as *Idh3a^−^.*

We bred compound heterozygous mice for *Idh3a*^−^ and *Idh3a^E229K^* and examined their eyes and visual function. As documented below, these mice have rapid retinal degeneration, demonstrating lack of complementation between the two alleles, and indicating that the E229K mutation is the cause of retinal degeneration in the mice found in the screen.

### Structural analysis of missense mutations

Residue Glu229 is located at the rim (Levy, 2010) of the heterodimeric interface formed between IDH3A and IDH3G (Fig. 1C), contributing only a small amount of surface area (9.7 Å^2^) to the much-larger interface (4401 Å^2^). However, molecular modelling of the E229K mutation, which replaces a negatively charged side chain with a positive charge, shows that it is likely to moderately disrupt the IDH3A:IDH3G interaction with little effect on the stability of the IDH3A monomer (Fig. 1D). In contrast, all five of the previously identified damaging IDH3A mutations are located away from the interface, either in the protein interior (A175V, M239T, P304H, M313T) or on the protein surface (R316C). All of these mutations are predicted to destabilise the IDH3A monomer, but not the interaction with IDH3G.

### Loss of *Idh3a* is lethal

As IDH3 is a critical part of the mitochondrial tricarboxylic acid (TCA) cycle, we asked whether the loss-of-function insertion allele was compatible with life. We intercrossed mice carrying the *Idh3a^−^* allele. No homozygous mice were recovered at weaning from a total of 120 genotyped, indicating that a complete loss of function of *Idh3a* is lethal. We examined embryos from intercrosses at different stages. No homozygous embryos were recovered at embryonic day (E) 10.5 or E12.5. At E8.5, only the remains of developmentally delayed embryos were recovered, indicating lethality to be early in development (Table 1). We confirmed loss of function of *Idh3a* by western blot analysis of *Idh3a^−/+^* and *Idh3a^−/E229K^* mutant mouse embryonic fibroblasts (MEFs), which showed a reduction in IDH3A protein when compared with *Idh3a^+/+^*, *Idh3a^E229K/+^* and *Idh3a^E229K/E229K^* cell lines (Fig. S1). Heterozygous littermates were aged to 1 year but no retinal degeneration was observed, indicating that one functional allele of the gene is sufficient (Fig. S2).

**Table 1.**
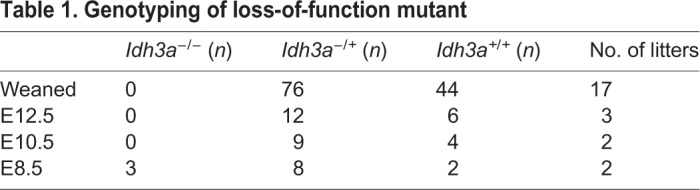
**Genotyping of loss-of-function mutant**

### Onset of retinal degeneration is early and rapid

**Fig. 2.**
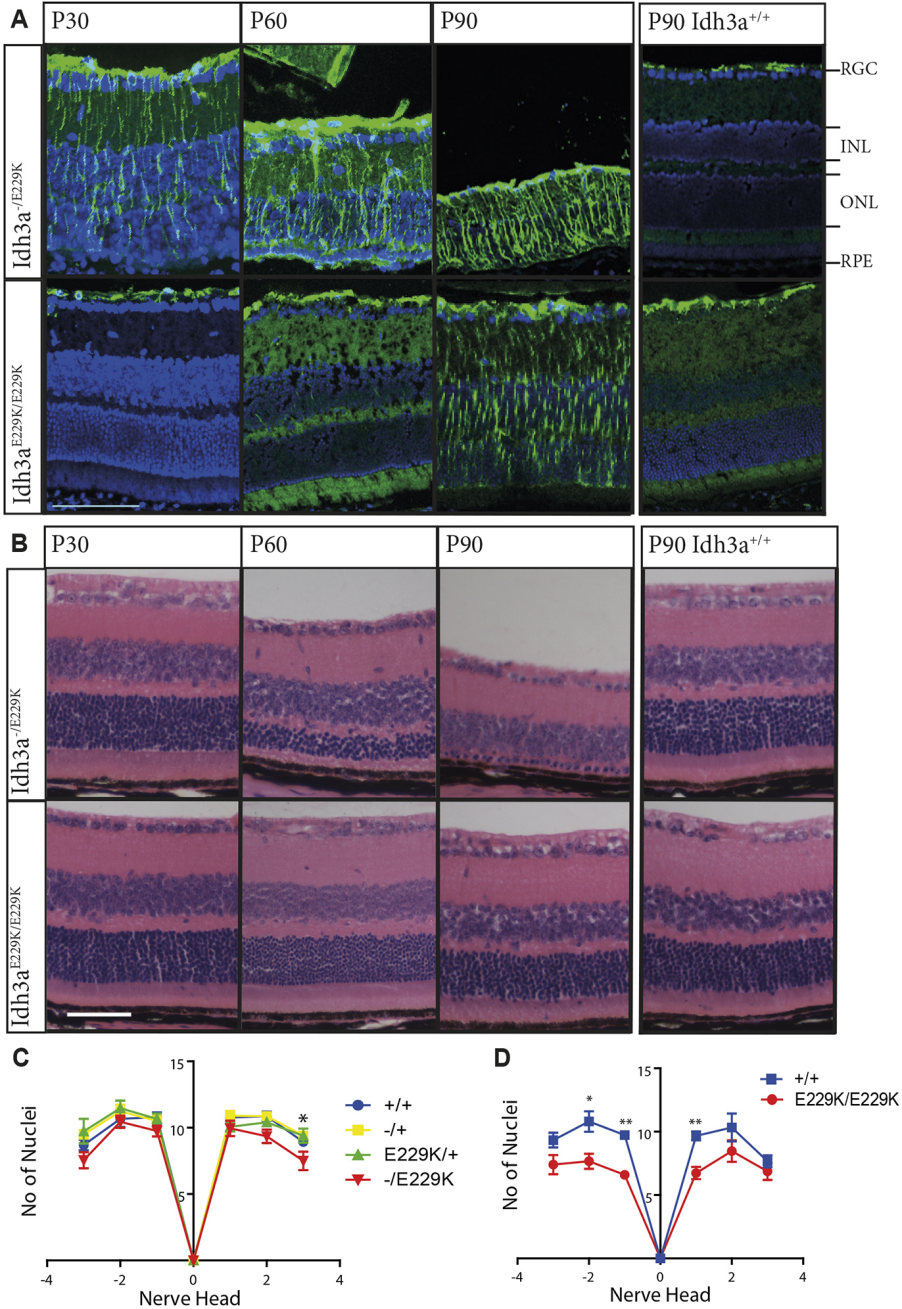
***Idh3a^−/E229K^* and *Idh3a^E229K/E229K^* mice show early and rapid photoreceptor loss.** (A) In *Idh3a^−/E229K^* mice, reactive gliosis, as indicated by green GFAP staining, is present from P30 and continues through P60-90 (*n*=3). This staining is not present in P30-60 *Idh3a^E229K/E229K^*, or in P90 wild-type littermates (rightmost), but is seen in P90 *Idh3a^E229K/E229K^* retinal sections (*n*=3). Blue staining is DAPI. (B) Histological analysis of H&E-stained eyes. The numbers of nuclei in the ONL are clearly decreased in *Idh3a^−/E229K^* at P60 and almost absent by P90. (C,D) Numbers of photoreceptor nuclei in the ONL (*y*-axis) at fixed distances either side of the optic nerve head (*x*-axis). Counts at P30 (C) show that all genotypes have normal nuclei numbers, except for the periphery of *Idh3a^−/E229K^* retinas (*n*=3-6, data in Table S2). By P60 and continuing to P90 (D), the numbers of nuclei are significantly decreased in *Idh3a^E229K/E229K^* retinas (*n*=7, F7, M2) compared with those of wild-type littermate controls (*n*=7, F4, M3). Unpaired *t*-test, *P<0.05, **P<0.01. Scale bars: 50 µm. INL, inner nuclei layer; ONL, outer nuclei layer; RGC, retinal ganglion cell layer; RPE, retinal pigmented epithelium.

We analysed the onset and progression of the retinal degeneration of both *Idh3a^E229K/E229K^* and *Idh3a^−/E229K^* mice by immunostaining for glial fibrillary acidic protein (GFAP), the expression of which is a sign of reactive gliosis of the Müller cells and an early indicator of stress within the retina (Eisenfeld et al., 1984). We also stained histological sections to assess retinal structure and integrity. From postnatal day (P) 30, *Idh3a^−/E229K^* mice exhibit GFAP staining throughout the retina (Fig. 2A), highlighting intermediate fibrils protruding from the ganglion cell layer to the outer nuclei layer (ONL). Nevertheless, at this stage, *Idh3a^−/E229K^* mice still retain the same number of nuclei in the ONL (the nuclei of the photoreceptors) as control littermates (Fig. 2C) throughout almost the entire retina, with only a slight decrease in the nasal periphery. However, by P60, the ONL has lost a significant portion of nuclei, indicating a loss of photoreceptor cells (Fig. 2B). This loss progresses further by P90 (Fig. 2B). No other cells within the retina appear to be lost during this degeneration.

By contrast, *Idh3a^E229K/E229K^* mice up to P60 show no signs of reactive gliosis or loss of cells (Fig. 2A). It is not until P90 that they begin to show GFAP staining (Fig. 2A), and the mice begin to lose photoreceptor nuclei (Fig. 2D). Again, no other cells appear to be lost during this time.

### Prior to the loss of photoreceptors, mice exhibit normal retinal function

**Fig. 3.**
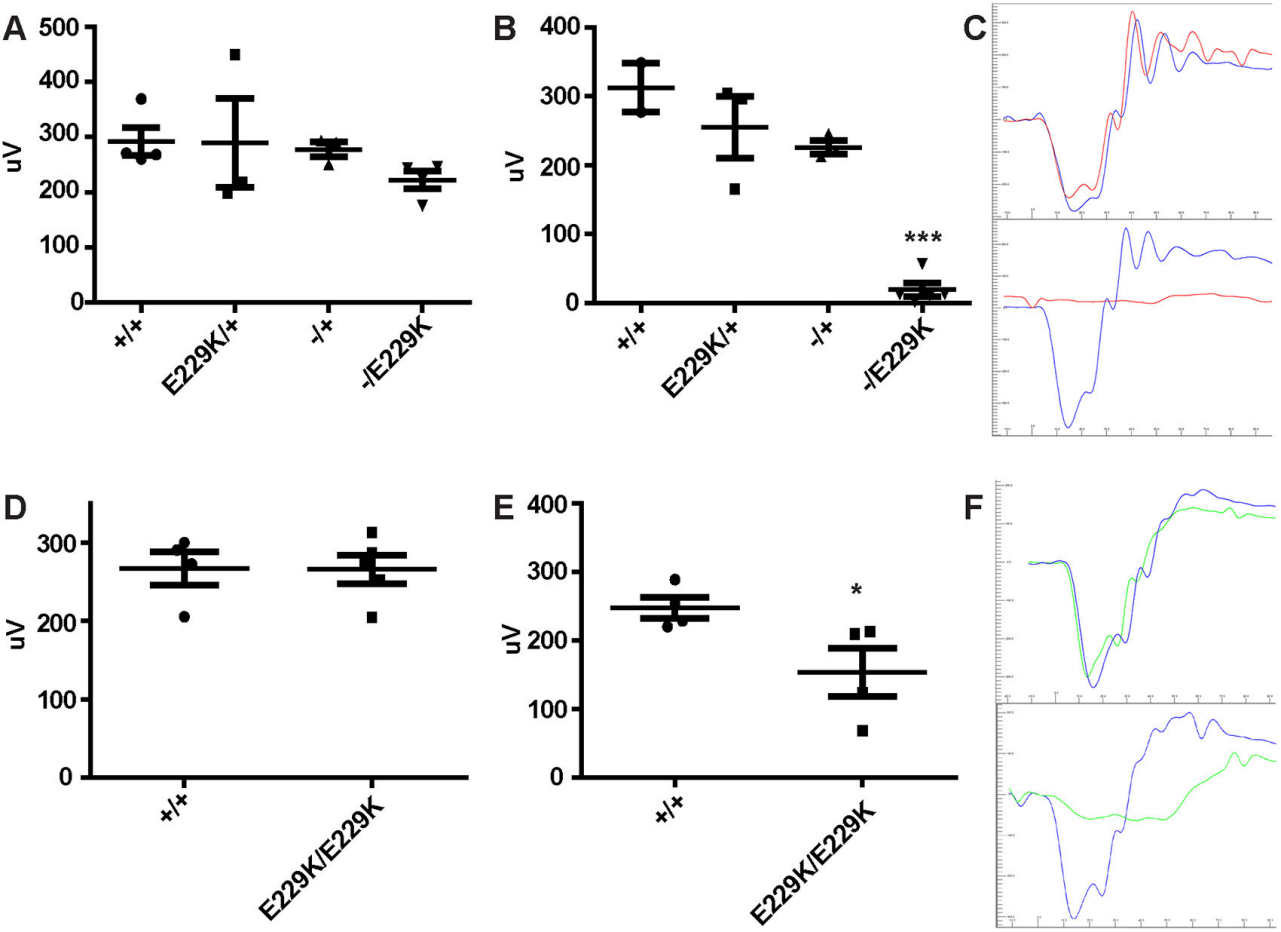
***Idh3a^−/E229K^* and *Idh3a^E229K/E229K^* mice show normal then decreased photoreceptor function.** (A-C) A-wave amplitudes of dark-adapted *Idh3a^−/E229K^* mice and littermate controls, exposed to a 3 cd/m^3^ flash of light at P30 (A) and P60 (B), with accompanying traces showing mutant (red) and wild-type (blue) scotopic responses at P30 (C, top) and P60 (C, bottom). At P30, *Idh3a^−/E229K^* mice exhibit a-wave amplitudes comparable to those of control littermates, as shown by one-way ANOVA [*F*(3,10)=0.7970, *P*=0.5232] [*n*=4 (F2, M2), 3 (F2, M1), 3 (F1, M2), 4 (F2, M2)], but by P60 this has significantly decreased [*F*(3,9)=32.66, ***P<0.001] [*n*=3 (M3), 3 (F1, M2), 3 (F1, M2), 5, (F1, M4)]. (D,E) Comparison of a-wave amplitudes between *Idh3a^E229K/E229K^* and wild-type controls at P60 (D) and P120 (E), respectively. At P60, mutant mice exhibit no loss of retinal function [265.5±18.18 (mean±s.e.m., *n*=5, F2, M3)] compared with wild-type littermates [267.0±21.3 (mean±s.e.m., *n*=4, F4)]. By P120, photoreceptor response to a light stimulus is significantly reduced in mutant [153.2±35.11 (mean±s.e.m., *n*=4, F2, M2)] compared with wild-type [247.7±15.51 (mean±s.e.m., *n*=4, F3, M1)] mice. *P<0.05. (F) Accompanying traces show *Idh3a^E229K/E229K^* (green) and wild-type control traces (blue) at P30 (top) and P120 (bottom).

We carried out electroretinogram (ERG) analysis of *Idh3a^−/E229K^* mice at P30. Although some reactive gliosis is detected at this stage, they nevertheless showed a normal response to light stimulus. A-wave amplitudes were not significantly different from those of wild-type and heterozygous (*Idh3a^−/+^* and *Idh3a^E229K/+^*) littermates, as shown by one-way ANOVA [*F*(3,10)=0.7970, *P*=0.5232], and the trace produced shows no abnormalities (Fig. 3A,C). The same mice tested at P60 showed a significantly reduced a-wave response, as shown by one-way ANOVA with Tukey post hoc tests [*F*(3,9)=32.66, *P*<0.0001], and mice exhibited a flatline ERG trace (Fig. 3B,C). The loss of a scotopic response is concurrent with the loss of photoreceptors observed in Fig. 2B.

The scotopic ERG responses of *Idh3a^E229K/E229K^* mice were tested at P60. Homozygous mutants showed no significant difference (*P*=0.9604) from wild-type littermates, showing a normal a-wave amplitude and trace (Fig. 3D,F). However, by 4 months of age, the a-wave amplitudes of scotopic ERG responses have significantly reduced in *Idh3a^E229K/E229K^* compared with those of wild-type littermates (*P*=0.0491, unpaired *t*-test with Welch's correction) (Fig. 3E,F).

### Mitochondria show a reduced maximal and reserve capacity

**Fig. 4.**
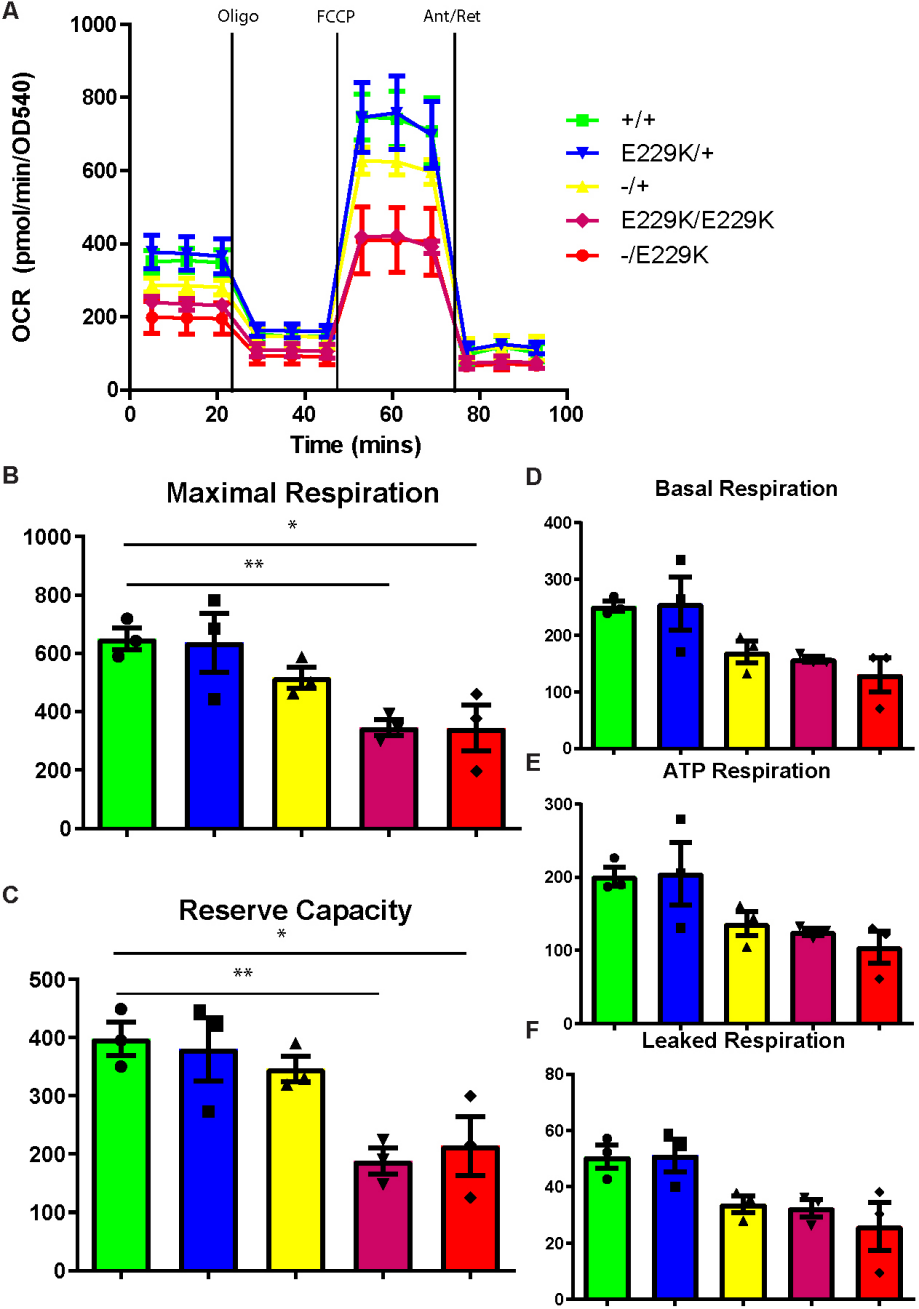
**Mutant MEF cells exhibit reduced reserve and maximal respiration.** *Idh3a* mutants exhibit reduced mitochondrial maximum respiration and reserve capacity. (A) Oxygen consumption (OCR) of MEF cells was measured using an Agilent Seahorse XF^e^24 Extracellular Flux Analyser in basal and stimulated conditions. (B-F) Histograms show maximal respiration [FCCP measurement 2 – antimycin+rotenone (Ant/Ret) measurement 2] (B), reserve capacity (FCCP measurement 2 – basal measurement 2) (C), basal respiration (basal measurement 2 – Ant/Ret measurement 2) (D), ATP respiration (basal measurement 2 – oligo measurement 2) (E), leaked respiration (oligo measurement 2 – Ant/Ret measurement 2) (F) pmol min^−1^ OD540^−1^ for each part of the experiment (A). *Idh3a^−/E229K^* and *Idh3a^E229K/E229K^* mutant cells show a lower, but not significant, trend in basal, ATP-dependent or leaked respiration compared with wild type (D-F). Both *Idh3a^−/E229K^* and *Idh3a^E229K/E229K^* showed significantly reduced maximum respiration [*F*(4,10)=5.701, *P*=0.0118] (B) and spare respiratory capacity [*F*(4,10)=6.536, *P*=0.0075] (C), as shown by one-way ANOVA. *P<0.05, **P<0.01.

As IDH3 is an essential part of the mitochondrial TCA cycle, we investigated how the *Idh3a* mutations affected the overall function of mitochondria (Fig. 4A). It has previously been shown (Kooragayala et al., 2015) that photoreceptors have a high oxygen demand and, as a consequence, their mitochondria are at maximum capacity. In order to measure the mitochondrial function in cells that are not already at maximum capacity, we derived multiple, independent fibroblast lines from *Idh3a^E229K/E229K^* and *Idh3a^−/E229K^* embryos, and heterozygous and wild-type littermates. When tested, the homozygous and compound heterozygous mutant cell lines showed a trend for lower basal (Fig. 4D), ATP (Fig. 4E) and leaked (Fig. 4F) respiration, although this trend was not statistically significant. However, both *Idh3a^E229K/E229K^* and *Idh3a^−/E229K^* cell lines did exhibit a significant decrease in maximal respiration (Fig. 4B) and spare respiratory capacity (Fig. 4C). Spare respiratory capacity (or reserve capacity) is a direct measure of a cell's ability to deal with respiratory stress. Mitochondrial dysfunction can result in cell death due to oxidative stress. We asked whether the mitochondrial defect in this case led to an excess of reactive oxygen species in the cells, and found no difference between mutant and wild-type cells (Fig. S3A, Table S3). Similarly, we measured the amount of mitochondrial proteins (Fig. S3B) and all cell lines showed similar amounts, indicating no change in mitochondrial number.

### *Idh3b* mutants do not exhibit the same phenotype as *Idh3a* mutants

As human patients with apparent loss-of-function mutations in *IDH3B* seem only to have retinal degeneration, we produced a CRISPR/Cas9-induced mutation in the mouse *Idh3b* gene. We identified an 8 bp deletion allele, c.478_487delCCAATGGA, and intercrossed carriers of this mutation, here abbreviated to *Idh3b^−^*. Homozygous animals were identified at normal Mendelian ratio.

**Fig. 5.**
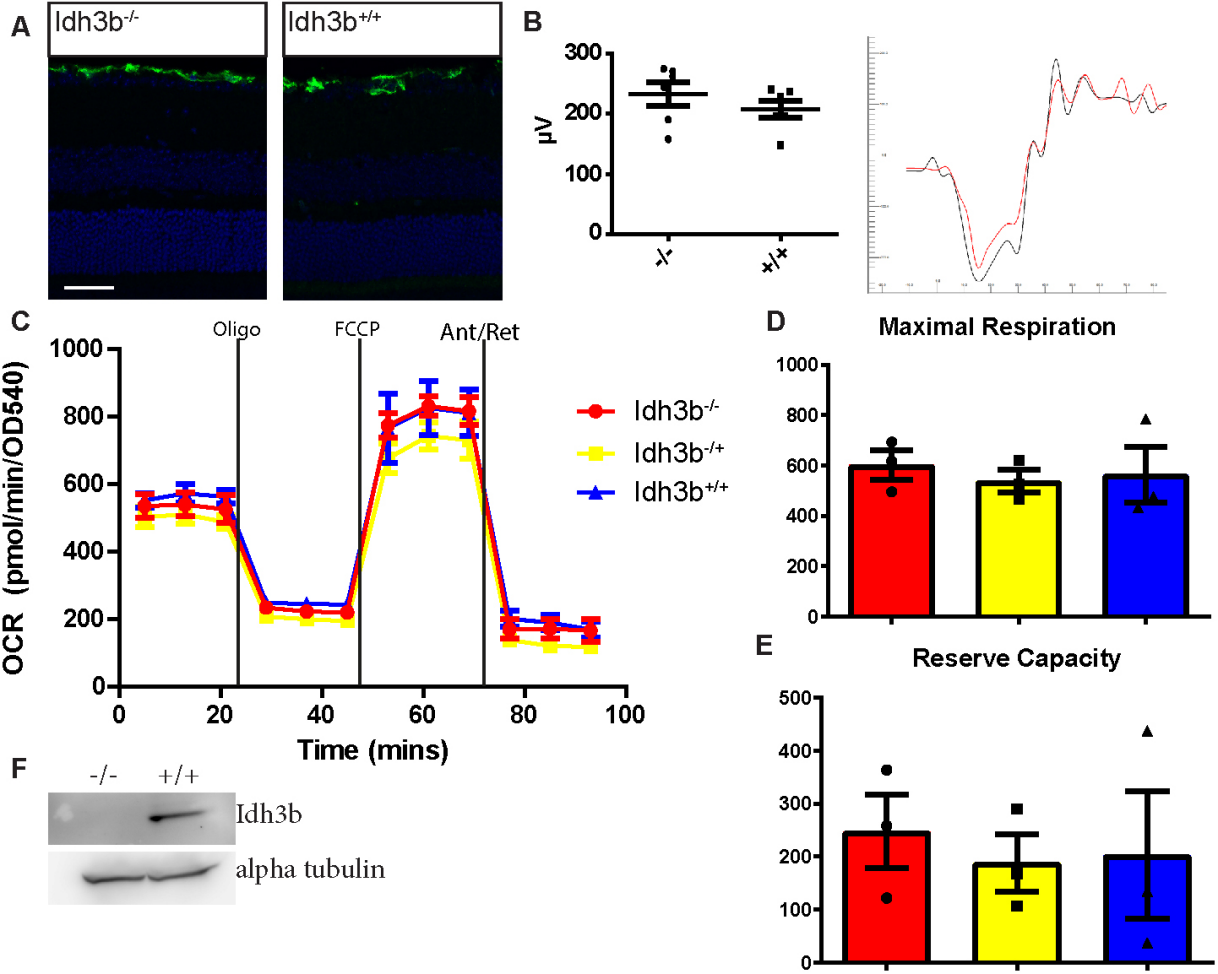
***Idh3b^−/−^* mice show no signs of retinal degeneration up to 6** **months.** *Idh3b*^−/−^ mice show no retinal degeneration up to P180. (A) GFAP green immunofluorescence on retinal sections shows no upregulation in mutant mice, indicating the absence of retinal stress (*n*=3). (B) ERG analysis of P180 *Idh3b^−/−^* in response to 3 cd/m^3^ flash under scotopic conditions shows a similar a-wave amplitude [233.4±19.63 (mean±s.e.m., *n*=6, F2, M4)] to that of wild-type littermates [207.7±14.42 (mean±s.e.m., *n*=6, F3, M3)] (left). ERG traces of mutant mice (red) also show no defects when compared with wild type (black) (right). (C-E) OCR was measured in *Idh3b^−/−^* MEF cells and compared with that of control littermates. In contrast to *Idh3a* mutant cells, *Idh3b^−/−^* cells did not show significantly reduced maximum respiration or spare respiratory capacity. (F) Western blot analysis for IDH3B and α-tubulin in retinal tissue shows the absence of IDH3B protein in *Idh3b^−/−^* retinas. Scale bar: 50 µm.

Protein extracts from homozygous livers were probed on immunoblots following electrophoresis and no protein could be detected, indicating that the mutation causes a loss of function (Fig. 5F).

*Idh3b^−/−^* mice were aged and analysed in the same manner as *Idh3a* mutant mice. By P180, we observed no signs of retinal degeneration. There was no observed upregulation of GFAP in the *Idh3b^−/−^* mice compared with wild-type littermate controls (Fig. 5A). ERG analysis showed that homozygous mutants exhibited normal scotopic responses (Fig. 5B). Extracellular flux analysis of fibroblasts produced from mutant embryos also showed that these mutant cell lines' mitochondria were not affected by the loss of *Idh3b*, with normal reserve capacity and maximum respiration levels (Fig. 5C-E).

## DISCUSSION

In this study, we have investigated the retinal degeneration in a mouse line identified in a recessive ENU screen. This is the first mouse model of IDH3A-related chronic retinal degeneration described, and closely mirrors the phenotype observed in patients. However, is not possible to model the pseudocoloboma of the macula seen in human patients in mice, as they lack a macula. Through the course of the investigation, we confirmed the genetic source of the phenotype to be a missense mutation in the *Idh3a* gene. The amino acid mutated in the *Idh3a^E229K^* mouse is within the highly conserved nucleotide-binding domain observed in both NAD- and NADP-dependent dehydrogenases of animals, including *Caenorhabditis elegans* and *Drosophila* (Pierrache et al., 2017). E229 is close to the amino acid critical for binding the divalent cation necessary for activity (D233), and is within a short segment of β-sheet (Ma et al., 2017b).

Mutation of such a highly conserved amino acid residue in an important part of the enzyme might suggest that it would cause complete loss of function, but the fact that the insertion (null) mutation is embryonic lethal indicates that the E229K allele is actually a partial loss-of-function mutation. The human mutations that have been identified in patients with RP presumably also include partial loss-of-function alleles. Two mutations are clear nulls: a frameshift and a stop-gain. Each of these is in a compound heterozygote with a likely partial loss-of-function mutation: M239C and R316H. One patient was homozygous for A175V, again presumed to be a partial loss of function. A further patient was compound heterozygous for M313T and P304H (Pierrache et al., 2017). The latter mutation was also identified as homozygous in a child with severe neurological defects in addition to retinal degeneration, and thus M313T is another likely partial loss-of-function allele. The three patients with one clear null allele included both who had macular pseudocoloboma in addition to RP, suggesting that it is a further reduction in IDH3A activity that results in the additional pathology. Notwithstanding the variation in severity of the mutations, all the mutated amino acids are completely conserved across animals from mammals to *Drosophila* and *C. elegans*. Structural modelling suggests that all five human mutations destabilise the protein, and thus we suggest that they cause severe loss, but not complete absence, of this catalytic subunit.

Mice compound heterozygous for the missense and insertion mutations exhibited a severe form of retinal degeneration, but have no other detectable abnormal phenotypes. A single copy of the *Idh3a^E229K^* allele provides sufficient IDH3 function for apparently normal development and physiology, except in the photoreceptors.

As previously reported (Kooragayala et al., 2015), photoreceptor cells operate at ∼70-80% of their mitochondria's maximal capacity, which means that they have little to no reserve capacity to use when under metabolic respiratory stress. *Idh3a^E229K/E229K^* and *Idh3a^−/E229K^* MEFs have a much lower maximal and reserve capacity compared with that of control cells. This lowered reserve capacity of mutant mitochondria, combined with the already decreased reserve capacity of photoreceptor cells, may explain the non-syndromic retinal degeneration phenotype observed. Much like the human patients previously reported, mice with the more severe allele combined with the missense allele exhibit a more severe form of degeneration, although extracellular flux analysis shows that cells have a similar decrease in reserve and maximal respiration.

By using MEF cells, the mitochondrial function of mutant mice was observed in an unstressed system. Although the mice showed no overt phenotype other than retinal degeneration, their mitochondria in MEFs exhibited a significant reduction in spare respiratory capacity. Reduction in spare respiratory capacity has previously been shown to induce cell death in both neurons and cardiomyocytes (Pfleger et al., 2015; Yadava and Nicholls, 2007). This reduction is thought to induce cell death either by increasing the amount of reactive oxygen species until a threshold is met and the cells succumb, or by causing a reduction in the amount of ATP produced by the electron transport chain, caused by a decrease in the availability of substrates (Sansbury et al., 2011; Yadava and Nicholls, 2007). Both of these could explain the degenerative phenotype observed in the *Idh3a* mutant mice, within which the catalytic subunit of the IDH3 enzyme has reduced or partial function.

The presence of more than one IDH enzyme within the mitochondria has been used as evidence for redundant function, and the viability of human patients apparently lacking IDH3B function led others to suggest that it is IDH2 that is the main TCA cycle enzyme in all tissues except the retina (Hartong et al., 2008). As we have shown that loss of *Idh3a* is lethal early in development, it is clear that IDH2 cannot compensate, and it is the difference between loss of the catalytic subunit and the regulatory subunit that that results in the different outcomes. Furthermore, we used RT-PCR to confirm the expression of *Idh2* in the mouse retina, as well as all three subunits of *Idh3* (Fig. S1), indicating that it is not lack of IDH2 in the retina that results in retinal degeneration. On the other hand, when *Idh3a* activity was knocked down in INS-1 832/13 beta cells, glucose-dependent production of α-ketoglutarate was greater than that when both *Idh3a* and *Idh2* were knocked down (MacDonald et al., 2013). This indicates that IDH2 can at least partially make up for the loss of IDH3 in this assay. A compensatory mechanism for loss of IDH3 potentially would be the conversion of NADPH, produced by IDH2, to NADH by the enzyme nicotinamide nucleotide transhydrogenase (NNT) (Hoek and Rydström, 1988). This NADH would then be able to participate in oxidative phosphorylation as normal. Furthermore, the gene encoding NNT is mutated in the C57BL6/J strain (Mekada et al., 2009). However, we bred the mice in this study with C57BL6/N, with a normal *Nnt* gene, before backcrossing and selecting study animals that did not have the mutant *Nnt* gene*.* The lethality of the *Idh3a* knockout allele indicates that NNT is unable to enable compensation *in vivo.*

In contrast to *Idh3a* mutants, *Idh3b* mutant mice are fully viable and show no signs of retinal degeneration within 6 months of age. Histology and ERG analysis show no significant loss of retinal structure or function. This is also shown in the results of extracellular flux analysis whereby *Idh3b* mutant MEFs do not show compromised mitochondrial activity. The human patients with clear null mutations in *IDH3B* develop RP in adulthood; the mutant mice might not live long enough to develop retinal degeneration, or this might simply reflect species differences. Species differences in response to enzymatic dysfunction could also, of course, impact the phenotype of the *Idh3a* mutations. The α subunit of IDH3, encoded by *Idh3a*, is responsible for the catalytic activity (Bzymek and Colman, 2007). The α subunit alone is inactive and requires the β and γ subunits, encoded by *Idh3b* and *Idh3g*. Dimers of αγ or αβ have activity, but vary greatly in their properties. *In vitro*, αγ dimers have 36% the specific activity of the heterotetramer, whilst αβ dimers have only 16%. Furthermore, αγ dimers are activated at low concentrations of ATP, whereas αβ dimers are inhibited. The substrate, isocitrate, activates both the heterotetramer and the αγ dimers, but has no effect on the αβ dimers (Ma et al., 2017b). We believe that the catalytic activity of the αγ heterodimers explains the lack, or perhaps delay, of a degenerative phenotype in *Idh3b* mutant mice. This also explains why loss-of-function *IDH3B* patients exhibit milder symptoms and at a much later age than patients with *IDH3A* mutations. In addition, the E229K mutation we describe here is likely to be interfering with the α/γ interaction, indicating that this is important for full activity.

Mutation of many different mitochondrial protein-coding genes can each lead to inherited diseases, with a range of phenotypes. Although there are some common phenotypic consequences, principally neurological manifestations, there is no clear pattern that can predict the outcome of a particular gene mutation (Munnich, 2008; Rustin et al., 1997). Even amongst genes of the TCA cycle, there is a range of consequences. Some mutations in the mitochondrial aconitase gene (*ACO2*), similar to *IDH3A*, can result in severe neurological disease, progressive optic atrophy and retinal degeneration (Metodiev et al., 2014), whilst other mutations in the same gene can give optic atrophy only. Progressive neurodegenerative disease also arises from mutations in the gene for the TCA cycle enzyme α-ketoglutarate dehydrogenase. Succinate dehydrogenase (*SDHA*) mutations not only can result in the severe neurological disorder Leigh Syndrome, but also in one extended family can cause dilated cardiomyopathy (Rustin et al., 1997). Mutations in the gene for another succinate dehydrogenase subunit (*SDHD*) are also a cause of encephalomyopathy (Rustin et al., 1997). Muscle involvement in addition to neurological disease is also seen in mutations of the malate dehydrogenase gene, *MDH2* (Ait-El-Mkadem et al., 2017).

The high-energy demand of the nervous system, and in particular the retina, makes these cells particularly sensitive to deficiencies in mitochondrial function. In our novel mouse models, we show that total loss of the TCA cycle enzyme IDH3 is early embryonic lethal, indicating that the mitochondrial enzyme IDH2 cannot compensate. However, partial loss of function, modelling human patients, results only in retinal degeneration. Modelling human patient mutations in which *Idh3b*, the regulatory subunit of IDH3, is absent does not result in retinal degeneration in mice within 6 months of age, reflecting the later onset of RP in the patients. The mechanism of cell death in this and other mitochondrial retinopathies is not fully understood, and it remains to be addressed whether therapeutic options exist.

## MATERIALS AND METHODS

### Mice

*Idh3a^mpc201H^*, denoted as *Idh3a^E229K^*, mice were produced in a recessive ENU screen at MRC Harwell (Potter et al., 2016). *Idh3a^em1Jkn^* mice were made by microinjection of nuclear CRISPR/Cas9 nickase vectors px335 (Addgene), together containing two guide RNAs (gRNAs) targeting exon 7 (Table 2) to minimise off-target effects. Sequencing identified a 5 bp insertion mutation, c.702_703InsGTACT, in the gene.

**Table 2.**
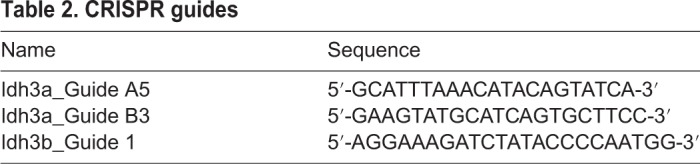
**CRISPR guides**

*Idh3b^em1Jkn^* mice were made by microinjection of CRISPR gRNA targeting exon 5 and Cas9 protein into the pronucleus of fertilised eggs (Table 2). Sequencing identified an 8 bp deletion mutation, c.478_487delCCAATGGA, in the gene. gRNAs were chosen with low off-target predictions and mice were backcrossed onto C57BL6/J prior to breeding. All animal work was approved by a University of Edinburgh internal ethics committee and was performed in accordance with the institutional guidelines under license by the UK Home Office.

### Immunohistochemistry

Mice were culled and eyes were enucleated and placed into Davidson's fixative for 1 h (cryosectioning) or overnight (wax embedding). For cryosectioning, eyes were removed from Davidson's fixative and placed into 10%, 15% and 20% sucrose in PBS for 15 min, 15 min and overnight, respectively. Eyes were then embedded using OCT cryopreservant and kept at −80°C until sectioning. For wax preservation, eyes were removed from Davidson's fixative and placed successively into 70%, 80%, 90% and 100% ethanol, xylene twice and then paraffin each for 45 min.

Haematoxylin and Eosin (H&E) staining was performed on 8 µm paraffin tissue sections, which were imaged on a Zeiss Axioplan 2 brightfield microscope. Anti-GFAP antibody (ab7260, Abcam) and Alexa Fluor 488 donkey anti-rabbit antibody (A21206, Invitrogen) were used on 8 µm frozen sections at 1:100 and 1:300, respectively. Slides were imaged on a Nikon A1R microscope.

### Generation of MEF cells

MEF cells were generated using E12.5-14.5 embryos as described in Takahashi and Yamanaka (2006), using Gibco Opti-MEM media (Thermo Fisher Scientific) with 10% fetal calf serum, and grown in hypoxic conditions (5% CO_2_, 3% O_2_). Cells were passaged three times before use, and littermate controls were used where possible.

### ERG

All mice undergoing an ERG were dark adapted for at least 2 h prior to the procedure, and experiments were carried out in a darkened room under red light using an HMsERG system (Ocuscience). Mice were anesthetised using isofluorane, and pupils were dilated through the topical application of 1% w/v tropicamide before being placed on a heated ERG plate. Three grounding electrodes were used subcutaneously (tail and each cheek) and silver-embedded electrodes were placed on the cornea, held in place with a contact lens. A standard ISCEV protocol was used, which recorded scotopic responses before a 10 min light adaption phase in order to record photopic responses. Scotopic responses of 3 cd.s/m^2^ light intensity were used for analysis.

### Extracellular flux analysis

Mitochondrial activity of MEF cells was analysed using the Seahorse Extracellular Flux Analyser, XF^e^24 (Agilent) as described in Lee et al. (2016). Cells were seeded onto a 24-well plate, at 15,000 cells per well, the day before analysis so cells were 90% confluent at the time of the assay (mutant cell lines appear to proliferate at the same rate as control cells). Oxygen consumption (OCR) was measured from cells in XF Assay medium supplemented with 10 mM glucose and 2 mM pyruvate (pH adjusted to 7.4 at 37°C). After three basal OCR measurements were obtained, OCR was measured following sequential injection of mitochondrial drugs (three measurements per drug injection); oligomycin (Port A, 1 µM), FCCP (Port B, 3.5 µM), antimycin and rotenone (Port C, 1 µM). Each measurement was derived from the rate of change in oxygen following a standard protocol (Mix, 3 min; Wait, 2 min; Measure, 3 min). Results were then standardised to cell number by sulforhodamine B assay, and results were reported by 540 absorption.

### Optokinetic reflex measurement

We assessed the visual acuity of the mice in the ENU screen using a virtual reality optokinetic system manufactured by Cerebral Mechanics, as described by Douglas et al. (2005). In brief, we observe the head-tracking response of the mouse to a variable density-moving grating. The mouse is positioned within a cabinet formed of monitors on which a virtual drum made of a vertical grating is generated by a computer. The grating density is varied to determine the point at which the head tracking stops, showing that the grating is no longer perceived.

### Structural analysis

The effects of the missense mutations on the crystal structure of the IDH3α:IDH3γ heterodimer (PDB ID: 5GRI) (Ma et al., 2017a) were modelled using the program FoldX (Guerois et al., 2002). The predicted effect on monomer stability (ΔΔG) was calculated with the FoldX BuildModel function, using the IDH3A subunit in isolation from the complex. The predicted change in interaction strength was calculated as the difference between the change in stability for the heterodimeric complex and that of the monomer.

### Western blot

Protein samples were ran on a 4-12% Tris-Bis gel (Invitrogen) and transferred to a PVDF membrane. Anti-α-tubulin (T5168, Sigma-Aldrich), anti-total OXPHOS (ab110413, Abcam), anti-IDH3A (15909-1-AP, Proteintech) and anti-IDH3B (ab121016, Abcam) were all diluted 1:1000 in PBS with 5% Marvel and incubated overnight at 4°C. Horseradish-peroxidase-conjugated anti-mouse (NA931V, GE Healthcare Life Sciences), anti-rabbit (NA934V, GE Healthcare Life Sciences) and anti-goat (sc-2020, Santa Cruz Biotechnology) antibodies were diluted 1:7000 in PBS with 5% Marvel and incubated at room temperature for 1 h.

### Reactive oxygen species detection

An Image-iT™ LIVE Green Reactive Oxygen Species Detection Kit (I36007, Invitrogen) was used as per the manufacturer's instructions. Cells were imaged using a Nikon A1R microscope, and analysis of fluorescent levels, normalised to 4′,6-diamidino-2-phenylindole (DAPI) fluorescent levels, was performed using ImageJ (https://imagej.nih.gov/ij/).

## Supplementary Material

Supplementary information
