# Supplementary material for: Mouse Idh3a mutations cause retinal degeneration and reduced mitochondrial function
Source: Dis Model Mech. 2018 Dec 18;11(12):dmm036426. doi: 10.1242/dmm.036426 (PMC6307916; doi:10.1242/dmm.036426)
Supplement: Supplementary information [file dmm-11-036426-s1.pdf]

Phenotyping Test	Parameter	Wild Type			Mutant			P value⁺
		Mean	SD	n	Mean	SD	n	
**Bodyweight (10w)**	**Males(g)**	32.17	4.80	46	27.92	5.07	5	0.148
	**Females (g)**	23.47	3.13	45	23.52	4.51	4	0.984
**Echo MRI (3 Months, Males)**	**Fat (g)**	8.7	3.23	45	5.8	2.70	5	0.076
	**Lean (g)**	24.5	3.01	45	22.5	4.47	5	0.382
	**Fat (%)**	23.2	6.21	45	18.0	5.41	5	0.101
	**Lean (%)**	67.5	5.42	45	72.2	6.49	5	0.194
**Bodyweight (22w)**	**Males(g)**	38.60	6.14	45	34.22	9.00	5	0.349
	**Females (g)**	35.69	5.90	45	35.37	7.07	4	0.936
**IPGTT (5 months, Males)**	**Glucose t=0 (mmol/l)**	10.2	2.51	45	8.88	1.41	5	0.115
	**Glucose t=60 (mmol/l)**	26.5	6.90	45	24.12	4.32	5	0.319
	**Glucose t=120 (mmol/l)**	17.3	5.40	45	13.68	3.08	5	0.058
	**Insulin t=0 (□g/l)**	1.0	0.40	45	0.875	0.24	5	0.348
**Clinical Chemistry (6 months, Females)**	**Urea (nmol/l)**	10.44	3.01	45	8.93	1.21	4	0.085
	**Creatinine (μmol/l)**	18.19	9.90	45	13.90	4.40	4	0.157
	**Calcium (mmol/l)**	2.40	0.13	45	2.43	0.06	4	0.439
	**ALP (U/l)**	89.73	19.52	45	84.40	10.82	4	0.435
	**ALT (U/l)**	31.18	5.11	45	29.25	2.65	4	0.262
	**AST (U/l)**	51.21	6.55	45	49.10	9.64	4	0.697
	**Albumin (g/l)**	33.60	1.93	45	34.90	1.52	4	0.208
	**LDH (U/l)**	371.99	86.54	45	402.95	68.41	4	0.459
	**Amylase (U/l)**	595.73	82.96	45	607.35	132.95	4	0.875
**Bodyweight (52w)**	**Males(g)**	46.14	7.85	40	42.72	8.68	5	0.449

	**Females (g)**	42.69	8.58	44	38.76	11.28	4	0.546
**Clinical Chemistry (12 months, Females)**	**Urea (nmol/l)**	9.07	2.34	45	10.80	2.54	4	0.280
	**Creatinine (µmol/l)**	13.79	6.70	45	21.65	13.90	4	0.345
	**Calcium (mmol/l)**	2.36	0.37	45	2.38	0.22	4	0.879
	**ALP (U/l)**	98.68	31.09	45	85.90	12.80	4	0.157
	**ALT (U/l)**	37.31	16.45	45	37.20	4.79	4	0.975
	**AST (U/l)**	61.89	21.77	45	64.95	24.42	4	0.824
	**Albumin (g/l)**	33.74	5.39	45	36.35	1.67	4	0.048*
	**LDH (U/l)**	411.96	220.92	45	877.90	773.73	4	0.316
	**Amylase (U/l)**	610.81	141.53	45	531.25	112.70	4	0.278
**Bodyweight (78w)**	**Males(g)**	47.20	7.69	22	41.31	6.36	4	0.175
	**Females (g)**	46.16	9.84	21	42.44	13.86	4	0.643
**Clinical Chemistry (18 months, Females)**	**Sodium (mmol/l)**	150.82	3.70	40	152.50	104.03	4	0.976
	**Potassium (mmol/l)**	6.32	1.81	40	7.78	3.19	4	0.434
	**Chloride (mmol/l)**	107.49	2.93	40	109.25	73.93	4	0.965
	**Urea (nmol/l)**	9.71	1.91	40	8.50	5.52	4	0.962
	**Creatinine (µmol/l)**	13.41	3.64	40	12.15	6.91	4	0.743
	**Calcium (mmol/l)**	2.42	0.23	40	2.41	1.55	4	0.991
	**Inorganic Phosphate (mmol/l)**	2.50	0.87	40	2.93	1.15	4	0.520
	**ALP (U/l)**	94.62	37.87	40	99.00	40.13	4	0.848
	**ALT (U/l)**	42.36	30.20	40	37.50	8.59	4	0.463
	**AST (U/l)**	83.87	46.77	40	70.50	26.23	4	0.415
	**Total Protein (g/l)**	53.20	3.09	40	53.18	35.43	4	0.999
	**Albumin (g/l)**	28.41	1.96	40	31.15	18.70	4	0.789
	**Total Cholesterol (mmol/l)**	4.40	0.96	40	3.73	2.43	4	0.622
	**HDL (mmol/l)**	2.86	0.58	40	2.48	1.61	4	0.671

Disease Models & Mechanisms • Supplementary information

	**LDL (mmol/l)**	0.91	0.34	40	1.00	0.40	4	0.693
	**Glucose (mmol/l)**	16.16	5.41	40	14.11	7.60	4	0.635
	**Triglycerides (mmol/l)**	1.95	0.75	40	1.87	0.85	4	0.868
	**Glycerol (µmol/l)**	574.51	222.14	40	685.00	249.17	4	0.456
	**Free Fatty Acids (mmol/l)**	1.22	0.38	40	1.69	0.60	4	0.222
	**Total Bilirubin (µmol/l)**	2.29	0.42	40	4.28	1.32	4	0.058
	**LDH (U/l)**	498.79	164.06	40	520.50	236.69	4	0.869
	**Amylase (U/l)**	608.15	86.25	40	521.75	369.04	4	0.672
	**Creatine Kinase (U/I**	169.54	160.17	40	219.00	6.63	4	0.060
	**Uric Acid (µmol/l)**	278.65	134.67	40	380.67	101.81	4	0.138
	**Fructosamine (µmol/l)**	228.62	44.81	40	173.25	129.97	4	0.459

Table S1 Phenotypic analysis of *Idh3a*$^{E229K/E229K}$ mice

No significant differences were observed between mutant mice and their wild type littermates apart from the 12 month Albumin concentration, however the mutant mice appear to be within normal levels and this was no longer significant by 18 months. $^{+}$Unpaired T-test with Welch's correction, * $p < 0.05$

Disease Models & Mechanisms • Supplementary information

Mouse	Sex	MPC	Idh3a	Periphery 1	middle 1	nerve 1	nerve2	middle 2	periphery 2
1002	M	WT	MUT	9	9.2	9.4	11.2	10.8	9.4
1003	M	MUT	MUT	9	10.6	8.8	9.4	8.2	6.2
1004	M	WT	WT	9	12.4	11.4	10	11.2	8.4
1005	M	WT	WT	9.6	11.2	10.2	11.4	11.2	9.4
1006	M	WT	MUT	9.4	11.6	10.6	11.6	10.6	8.6
1007	F	MUT	MUT	6.2	9.2	9.4	8.6	8.8	6.4
1008	F	WT	WT	7.2	9.6	10.8	11.2	11.2	8.8
1009	F	WT	WT	9.4	11.4	9.8	10.4	11.4	9.8
1010	F	WT	MUT	8.8	11.6	10	9.2	11.4	9
1202	M	MUT	WT	11.2	12.4	10	10.2	10	10.2
1203	M	MUT	WT	8	11.6	11.4	9.4	10.4	9.6
1204	M	WT	MUT	11	12.4	10	11.4	11.6	10.2
1205	M	MUT	MUT	8	11.4	10.2	11.2	10.4	8.6
1206	F	WT	MUT	9.2	12.2	12.8	11.4	10.4	9
1207	F	WT	MUT	9.6	10.4	10.2	10.6	10.4	10.2
1208	F	WT	WT	9.2	11.2	12.2	10.6	9.2	8.2
1209	F	MUT	MUT	7	10.6	10.6	10.6	10	8.8
1210	F	WT	WT	7.8	8.2	10.4	11	11	9
1211	F	MUT	WT	10	10.4	10.6	10.6	10.8	8.4

Table S2. Outer nuclear layers of offspring of Idh3a $^{E229K/-}$ intercross. WT and MUT indicate wild-type or mutant for the particular allele. Number of nuclei in sections through the outer nuclear layer. Each is the mean of 5 counts (technical replicates).

Disease Models & Mechanisms • Supplementary information

Genotype	Average Fluorescence Intensity			
	**Cell line 1**	**Cell line 2**	**Cell line 3**	**Cell line 4**
+/+	203.128	263.989	264.796	278.359
E229K/+	322.033	294.909	327.883	
-/+	292.315	262.582	250.29	258.325
E229K/E229K	230.348	272.818	265.045	222.117
-/E229K	360	216.375	267.474	

Table S3 **Reactive Oxygen Species**: Data for Figure S3. 3 or 4 cell lines (biological replicates) from each genotype were analysed with measurements in 4 wells (technical replicates). The means of the technical replicates are shown for each biological replicate.

Disease Models & Mechanisms • Supplementary information

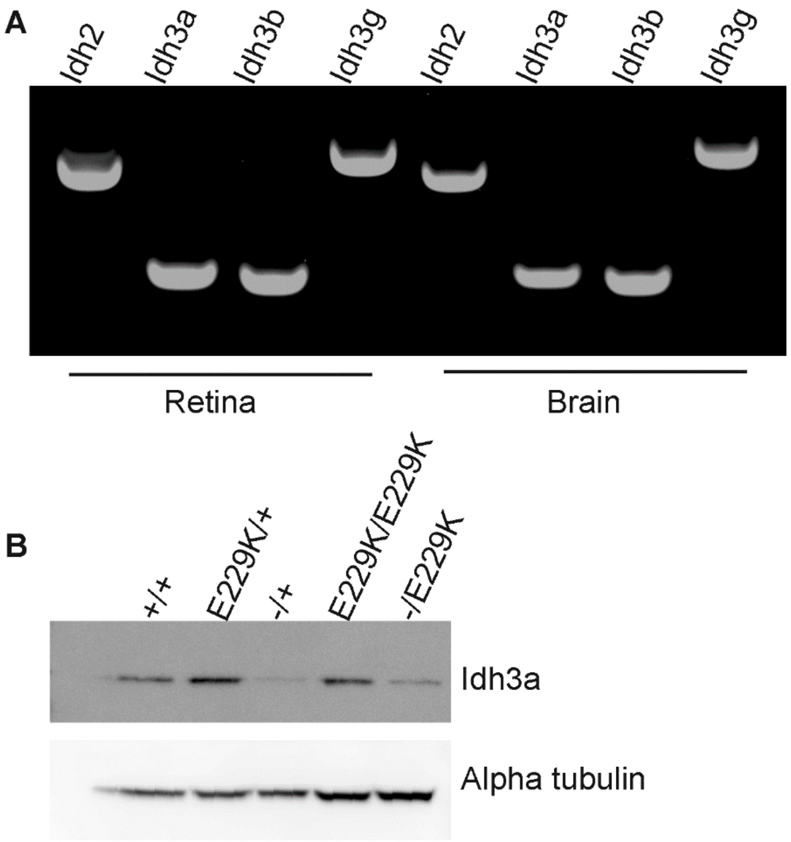

Figure S1 Analysis of *Idh3a* expression and protein levels in mutant MEF cells

(A) RNA was isolated from C57BL6/J mice retina and brain and RT-PCR was performed to assay the expression of *Idh3* heterotetramer components and *Idh2* genes. All were present in all samples. **(B)** Western blot showing the reduced protein in *Idh3a*$^{-/+}$ and *Idh3a*$^{-/E229K}$ compared to control littermates (*Idh3a*$^{+/+}$ and *Idh3a*$^{E229K/E229K}$) as well as *Idh3a*$^{E229K/E229K}$ mutant cells. Alpha-tubulin was used as a loading control.

Disease Models & Mechanisms • Supplementary information

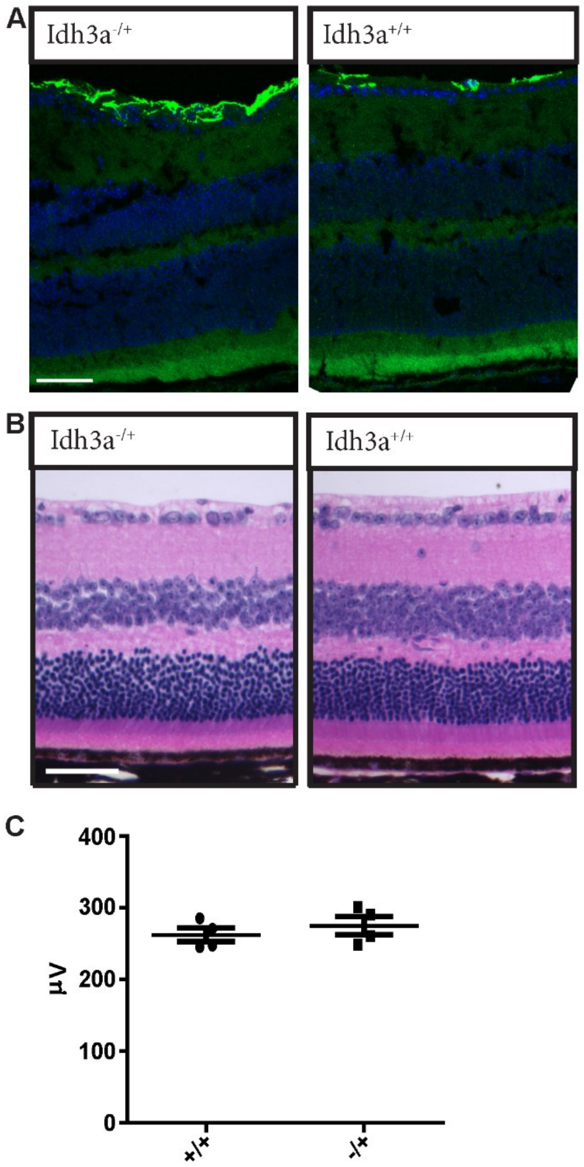

Disease Models & Mechanisms • Supplementary information

Figure S2 Analysis of *Idh3a*$^{-/+}$ mice show no retinal phenotype up to one year

(A) *Idh3a*$^{-/+}$ mice show no upregulation of GFAP at one year, with staining comparable to that of wild type littermates (n = 3). **(B)** Morphology of retinal layers, including the outer nuclear layer, appears normal at 12 months and is comparable to that of wild type littermates (n = 6). **(C)** *Idh3a*$^{-/+}$ mutant mice show no reduction in retinal function at one year, exhibiting similar ERG a-wave responses to those of wild type littermates ($p = 0.286$, n = 4).

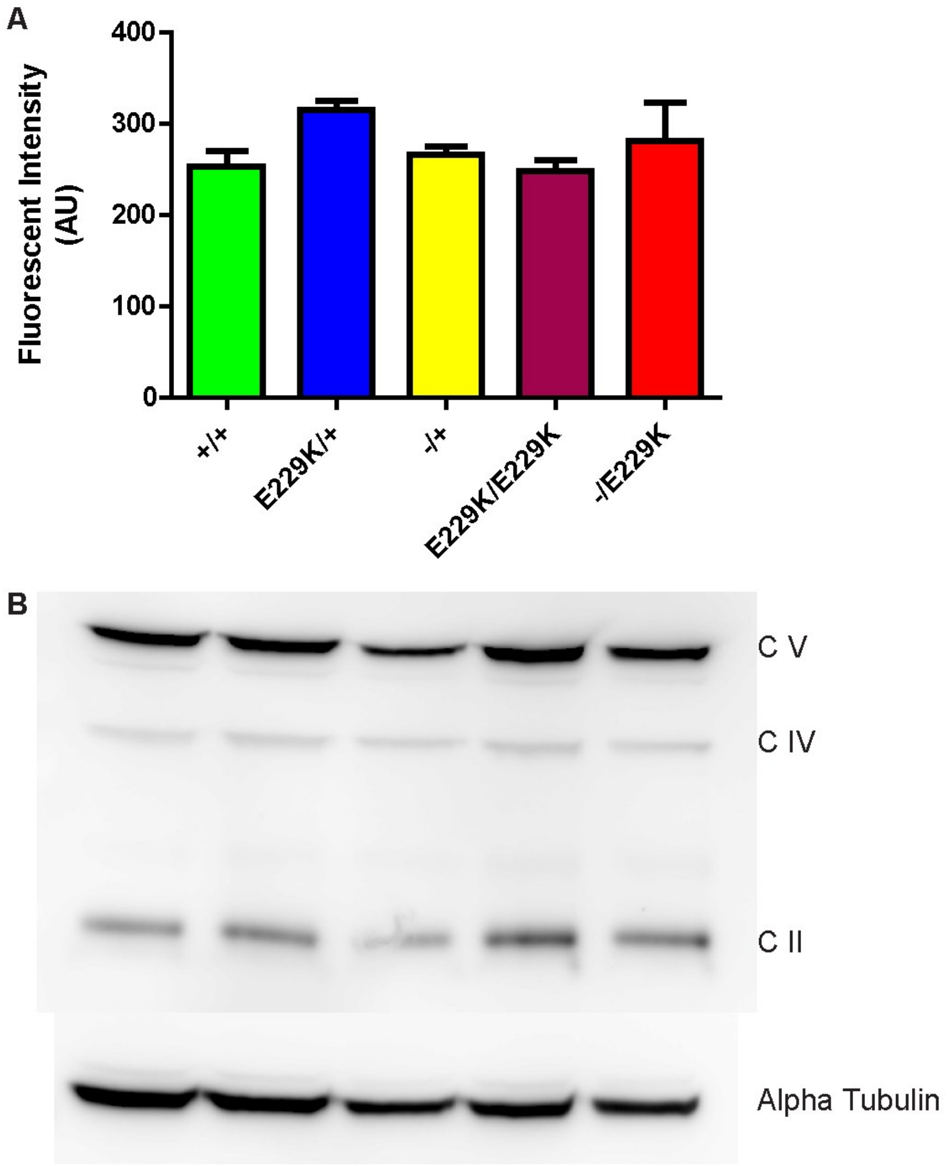

Disease Models & Mechanisms • Supplementary information

Figure S3 Further analysis of MEF cell lines

(A) Reactive Oxygen Species (ROS) were measured in each of the cell lines, both *Idh3a*$^{-/E229K}$ and *Idh3a*$^{E229K/E229K}$ cells showed levels similar to that of control cells (*Idh3a*$^{+/+}$, *Idh3a*$^{-/+}$ and *Idh3a*$^{E229K/+}$), graph shows fluorescent intensity in arbitrary units Data is in Table S3. **(B)** Western blot analysis of MEF cell lines showing similar levels of mitochondrial proteins; Complex II, Complex IV and Complex V. Alpha-tubulin was used as a loading control.
